# *six3* acts upstream of *foxQ2* in labrum and neural development in the spider *Parasteatoda tepidariorum*

**DOI:** 10.1007/s00427-020-00654-9

**Published:** 2020-02-10

**Authors:** Magdalena Ines Schacht, Christoph Schomburg, Gregor Bucher

**Affiliations:** 1 Department of Evolutionary Developmental Genetics, GZMB, University of Göttingen, Justus-von-Liebig-Weg 11, 37077 Göttingen, Germany; 2 School of Biological and Chemical Sciences, Queen Mary University of London, Mile End Road, London, E1 4NS UK; 3 Institut für Allgemeine Zoologie und Entwicklungsbiologie, Justus-Liebig-Universität Gießen, Heinrich-Buff-Ring 38, 35392 Giessen, Germany

**Keywords:** Spiders, Labrum, RNAi, Gene interaction

## Abstract

Anterior patterning in animals is based on a gene regulatory network, which comprises highly conserved transcription factors like *six3, pax6* and *otx*. More recently, *foxQ2* was found to be an ancestral component of this network but its regulatory interactions showed evolutionary differences. In most animals, *foxQ2* is a downstream target of *six3* and knockdown leads to mild or no epidermal phenotypes. In contrast, in the red flour beetle *Tribolium castaneum*, *foxQ2* gained a more prominent role in patterning leading to strong epidermal and brain phenotypes and being required for *six3* expression. However, it has remained unclear which of these novel aspects were insect or arthropod specific. Here, we study expression and RNAi phenotype of the single *foxQ2* ortholog of the spider *Parasteatoda tepidariorum.* We find early anterior expression similar to the one of insects. Further, we show an epidermal phenotype in the labrum similar to the insect phenotype. However, our data indicate that *foxQ2* is positioned downstream of *six3* like in other animals but unlike insects. Hence, the epidermal and neural pattering function of *foxQ2* is ancestral for arthropods while the upstream role of *foxQ2* may have evolved in the lineage leading to the insects.

**Electronic supplementary material:**

The online version of this article (10.1007/s00427-020-00654-9) contains supplementary material, which is available to authorized users.

## Introduction

Anterior patterning in animals is based on an anterior gene regulatory network (aGRN), which is built by a conserved set of transcription factors and signalling pathways (Lowe et al. 2003; Marlow et al. 2014; Posnien et al. 2011; Sinigaglia et al. 2013; Steinmetz et al. 2010; Yaguchi et al. 2008; Tosches and Arendt 2013). However, functional studies revealed that the regulatory interactions within this ancestral patterning system have diverged between animals (Range and Wei 2016; Sinigaglia et al. 2013; Yaguchi et al. 2008; Kitzmann et al. 2017). One example is the function of the forkhead box transcription factor *foxQ2*. The forkhead box (Fox) family is a large family of transcription factors that holds many important regulatory roles in numerous developmental and homeostatic processes (Fritzenwanker et al. 2014; Uhlenhaut and Treier 2011; Benayoun et al. 2011; Carlsson and Mahlapuu 2002), and is widely represented in the animal kingdom (Kaestner et al. 2000). *foxQ2* is a member of this transcription factor family, which is present in all major lineages of the metazoans (Chevalier et al. 2006; Sinigaglia et al. 2013; Marlow et al. 2014; Tu et al. 2006; Yu et al. 2003; Lee and Frasch 2004). In deuterostomes, such as the cephalochordate *Branchiostoma floridae* (*B. floridae*) and the echinoderm *Strongylocentrotus purpuratus* (*S. purpuratus*), *foxQ2* is expressed at the anterior pole from the late blastula stage (*B. floridae*) or mesomeric cleavage stage (*S. purpuratus*) onwards. In both cases, transcripts are still detected in larval stages in the anterior tip of the ectoderm (Yu et al. 2003; Tu et al. 2006; Yaguchi et al. 2008; Yankura et al. 2010; Range and Wei 2016). The three paralogs of *foxQ2* in the hemichordate *Saccoglossus kowalevskii* (*S. kowalevskii*) also share a largely apical expression pattern, while exhibiting temporal differences (Fritzenwanker et al. 2014). A conserved apical expression of *foxQ2* is also present in lophotrochozoans, such as the annelid *Platynereis dumerilii* (*P. dumerilii*) and the brachiopod *Terebratalia transversa* (*T. transversa*) (Santagata et al. 2012; Marlow et al. 2014), as well as in the early development of the insects *Drosophila melanogaster* (*D. melanogaster*) and *Tribolium castaneum* (*T. castaneum*) (Lee and Frasch 2004; Kitzmann et al. 2017). Additionally, in non-bilaterian species, such as the cnidarians *Nematostella vectensis* (*N. vectensis*) and *Clytia hemispherica* (*C. hemispherica*), *foxQ2* expression is restricted to the aboral pole of the embryos (Chevalier et al. 2006; Sinigaglia et al. 2013), supporting the hypothesis of homology between the cnidarian aboral pole and the bilaterian anterior pole (Sinigaglia et al. 2013). Interestingly, while orthologs are found in fish, the *foxQ2* gene was apparently lost from the genomes of amphibians and mammalians.

*foxQ2* is expressed in neurogenic regions of different species (Kitzmann et al. 2017; Hunnekuhl and Akam 2014; Marlow et al. 2014; Santagata et al. 2012). In *P. dumerili*, *foxQ2* and *six3* are expressed in the apical plate including sensory-neurosecretory cells (Marlow et al. 2014). Similarly, in the myriapod *Strigamia maritima* (*S. maritima*), nested domains of *foxQ2* and *six3* expression were identified in neural precursors of the embryonic head (Hunnekuhl and Akam 2014). Both these transcription factors pattern anterior neurogenic domains during the embryonic development of the brachiopod *T. transversa* (Santagata et al. 2012). In the red flour beetle, *Tc-foxQ2*-positive cells contribute to the central complex and the gene is required for central brain development (He et al. 2019). In *T. castaneum*, an extended GRN for anterior head development has been proposed in which *Tc-six3* and *Tc-foxQ2* hold upstream positions (Kitzmann et al. 2017). They mutually regulate each other forming an upstream core unit, which regulates several downstream targets. Those targets are required for the proper formation of anterior structures, like the head placode, neurogenic ectoderm, labrum and stomodeum, and are therefore important for the development of the head and brain (Kitzmann et al. 2017). Intriguingly, no or only mild epidermal effects were found in *foxQ2* knockdown embryo in other animals, while in *T. castaneum*, a strong effect on epidermal patterning was observed (Kitzmann et al. 2017). This finding correlated with the downstream role of *foxQ2* in most analysed species except for *T. castaneum*. However, it has remained unclear which of these aspects are ancestral for arthropods and which may be insect specific.

In order to gain insights from a basal euarthropod, we studied the *foxQ2* expression, its RNAi phenotype and its interaction with *six3* in the spider *P. tepidariorum*. We find that the epidermal labrum phenotype is conserved but that spider *Pt-foxQ2* appears not to regulate either of the two *Pt-six3* paralogs.

## Material and methods

### Phylogenetic analysis

The *Pt-foxQ2* sequence (aug3.g4586.t1) was identified by performing a BLAST search using the translated nucleotide query of FoxQ2 from *D. melanogaster* (declared on FlyBase as fd102C, FlyBase ID: FBgn0039937) in the *P. tepidariorum* genomic gene predictions (Schwager et al. 2017). The 20 best-scoring sequences were combined with published protein sequences of FoxQ2, as well as the protein sequence of FoxP of *D. melanogaster* and *Danio rerio*, which were retrieved from Fritzenwanker et al. (2014). All protein sequences were subsequently used to perform a protein alignment with default settings using Clustal Omega (Sievers et al. 2011; Sievers and Higgins 2014). Phylogeny was inferred based on this alignment using MrBayes 3.2.0 (Ronquist et al. 2012), which was sampled from a total of 1836 runs using the Wag model of amino acid substitution.

### Animal culture and gene cloning

Embryos and adults of *P. tepidariorum* were obtained from the stock in Göttingen. Spider were kept and fed as previously described (Turetzek et al. 2015; Pechmann et al. 2017). Embryos used for in situ hybridization (ISH) experiments were staged, collected and fixed as previously described (Hilbrant et al. 2012; Mittmann and Wolff 2012; Posnien et al. 2014). The *P. tepidariorum* genes *six3.1* (NCBI GenBank: AB605265.1) and *six3.2* (NCBI GenBank: AB605266.1) used in this work were already published in Schomburg et al. (2015). The identified *Pt-foxQ2* sequence (NCBI GenBank: MN567069) was amplified from cDNA (fwd primer: ^5’^CGCTACTGACCAGAACCCTTT^3’^, rev primer: ^5’^TAAGGAAGCAGCGGATGACCA^3’^ amplified a 979 bp long sequence) and cloned into the pJet1.2 vector (Thermo Fisher Scientific).

### Probe synthesis and in situ hybridization

*Pt-foxQ2* antisense mRNA probes for ISHs were synthesized from the entire cloned sequence (979 bp) with the DIG RNA labelling Mix using the T7 RNA polymerase (both Roche, DIG RNA labelling Mix with Cat.-No.: 11277073910 and T7 Polymerase with Cat.-No.: 10881767001). The *Pt-six3.1* and *Pt-six3.2* antisense mRNA probes were those used in Schomburg et al. (2015) and are of 1439 bp and 1328 bp length, respectively. ISHs and nuclear SYTOX® Green staining in *P. tepidariorum* embryos were carried out as described previously (Prpic et al. 2008; Pechmann and Prpic 2009).

### dsRNA synthesis and RNA interference

The complete cloned sequence for *Pt-foxQ2*, *Pt-six3.1* and *Pt-six3.2* (see details above) was amplified using vector specific primers combined with the T7 promoter sequence (^5’^GAATTGTAATACGACTCACTATAGG^3’^) in standard PCR and served as a template for the in vitro transcription. Double stranded RNA (dsRNA) of each gene was synthesized using the Ambion® T7 MEGAscript® Kit (Life Technologies, Carlsbad, CA, USA). Precipitation of the transcribed dsRNAs and the injection procedure was performed as described previously (Akiyama-Oda and Oda 2006; Turetzek et al. 2015). For *Pt-foxQ2*, *Pt-six3.1* and *Pt-six3.2*, 2.5 μl of dsRNA (4 μg/μl) was injected into sexually mature adult female spiders using borosilicate injection needles. Females were treated with 5 injections in total, whereby females were injected every second day and mated after the third injection. The same injection regime was applied for the negative control spiders that were injected with injection buffer only. Previous studies used GFP as a control injections which result in very few unspecific effects in RNAi embryos (Schwager et al. 2009; Paese et al. 2018). Therefore, we decided to use injection buffer only. Each female was kept and fed in the same way. The first four cocoons of each spider were collected, opened and kept in test tubes provided with a damp tissue to maintain humidity, essential for proper development. Half of each cocoon was fixed, whereas the other half could develop. During development, the embryos were checked regularly under the stereomicroscope and individual embryos of each cocoon were analysed in Voltalef® oil. All embryos were screened for any visible phenotypes. For the analysis of the embryos, batch 2–4 were merged because they had similar strong effects while batch 1 showed less penetrance.

### Microscopy and imaging

Images of *P. tepidariorum* embryos were taken with a Leica M205 FA binocular (Leica Microsystems, Wetzlar, Germany) and recorded with the ImagePro v.7.0 software. Images were taken with fluorescent and white light simultaneously. After white correction, the green SYTOX® appears light blue, while the DIG staining appears dark blue, thus allowing staining and morphology to be shown in the same picture. Images were corrected for colour values and brightness with the Adobe Photoshop® CS6 software (version 9.0).

## Results

### *P. tepidariorum* possesses one homolog of *foxQ2*

**Fig. 1:**
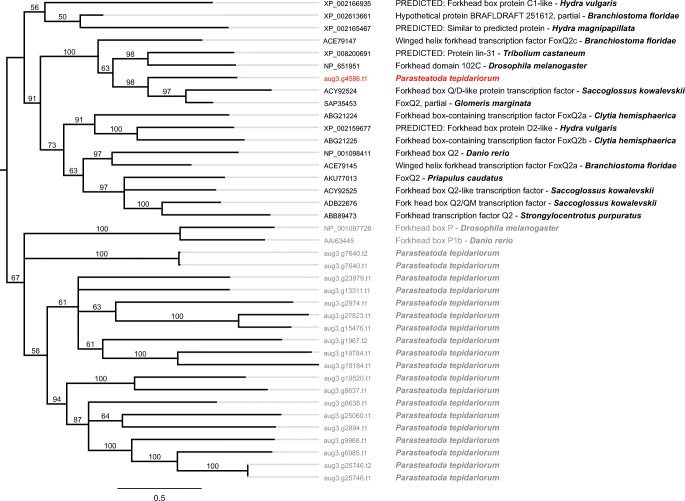
Phylogenetic tree showing the *P. tepidariorum* FoxQ2 homolog. The *P. tepidariorum* predicted protein aug3.g4586.t1 (shown in red) clusters with the other FoxQ2 sequences and no other paralog was identified in this spider. Number on branches indicates posterior probabilities as calculated by MrBayes

Large-scale duplication of genes in spiders has been reported in *P. tepidariorum* before (Pechmann et al. 2015; Turetzek et al. 2015; Schomburg et al. 2015; Schwager et al. 2017; Leite et al. 2018). Therefore, we wanted to identify the total number of *foxQ2* orthologs in *P. tepidariorum*. We performed a multiple sequence alignment with FoxQ2 protein sequences of different metazoan species and generated a phylogenetic tree based on Bayesian analysis. The phylogenetic tree (Fig. 1) shows that the spider’s translated transcript aug3.g4586.t1 clusters with all FoxQ2 sequences from the different species and is therefore indeed the spider *foxQ2* ortholog. None of the other identified *P. tepidariorum* sequences cluster with *Pt-*FoxQ2 or any other analysed metazoan FoxQ2 sequence, indicating that there is only one homolog of *foxQ2* in the spider (shown in red, Fig. 1).

### *Pt-foxQ2* shows conserved expression in the anterior region of the embryo

**Fig. 2:**
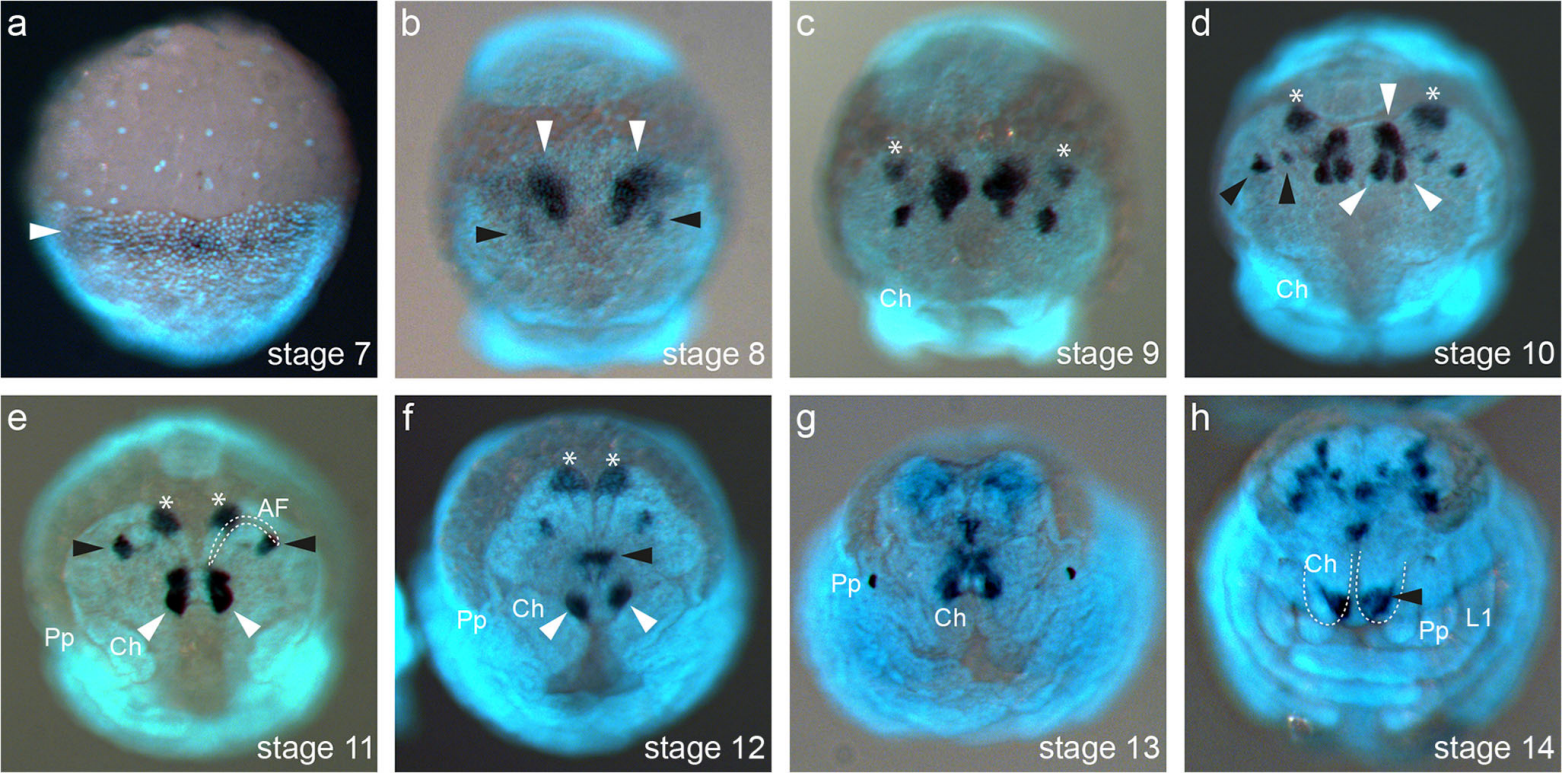
Embryonic *Pt-foxQ2* expression. After initial expression as a stripe at the anterior end of the embryo (white arrowhead in a), *Pt-foxQ2* is found in several dynamic domains in the pre-cheliceral lobes (**b**–**f**). White and black arrowheads and asterisks mark the corresponding domains in all panels. From stage 11 onward, the head epidermis grows over the neurogenic ectoderm (anterior furrow (AF) in **e**). At later stages, *Pt-foxQ2* transcripts are found in the labrum and the brain (**g**, **h**). Expression is not in the chelicerae but below of them (black arrowhead in **h**). All embryos shown in frontal view. Developmental stages are indicated in the lower right-hand corner. AF: anterior furrow; Pp: pedipalps; Ch: chelicerae; L1: first walking leg)

Previous studies in different metazoan species showed that *foxQ2* is expressed in the anterior regions during embryonic development (Yu et al. 2003; Lee and Frasch 2004; Tu et al. 2006; Marlow et al. 2014; Fritzenwanker et al. 2014; Kitzmann et al. 2017) but expression analysis of *foxQ2* in chelicerates had been missing. Here, we analysed the expression of *Pt-foxQ2* during spider embryogenesis (see Mittmann and Wolff, 2012 for detailed description of morphology during embryogenesis and Schomburg et al. 2015 for description of anterior structures for embryonic stages 10 to 13). We found that expression of *Pt-foxQ2* started at stage 7, when the germ band became evident, spanning the anterior rim of the germ band as a broad stripe (arrowhead in Fig. 2a; see Fig. S1 for stages 3–6, devoid of *Pt-foxQ2* staining). At stage 8, *Pt-foxQ2* was bilaterally expressed in the anterior part of the pre-cheliceral region in one larger domain (white arrowheads in Fig. 2b) and an emerging smaller domain posterior-laterally to it (black arrowheads in Fig. 2b). Expression of *Pt-foxQ2* showed a tripartite domain on each side of the pre-cheliceral region at stage 9 with an additional domain on both sides of the anterior non-neurogenic ectoderm (asterisks in Fig. 2c; the non-neurogenic ectoderm in the pre-cheliceral lobes is all ectoderm along the rim of the head lobes outside of the anterior and lateral furrows. This area is free of neural precursor invagination sites—see Fig. S2 for a scheme of a stage 11 embryo and Schomburg et al. 2015 for extensive description from stage 10 to 14). At stage 10, these three domains were localized in a different pattern. Transcripts of *Pt-foxQ2* in the non-neurogenic ectoderm became localized more anteriorly (asterisks in Fig. 2d–f), while the median domains split into three distinct domains (white arrowheads in Fig. 2d). The lateral expression in the neurogenic ectoderm splits into two domains between the edge of the head lobes and the prospective labrum (black arrowheads in Fig. 2d). At stage 11, the most lateral expression domains remained at the end of the anterior furrow (black arrowheads in Fig. 2e), while the median expression domain shifted posteriorly along with the stomodeum and labrum. The former domains fused into one (white arrowheads in Fig. 2e). These domains split up again at stage 12 with one part remaining in the region of the labrum (black arrowhead in Fig. 2f), while the other shifted further towards the posterior at stage 12 (white arrowheads in Fig. 2f). During stage 13, the non-neurogenic ectoderm started to grow over the brain region (Fig. 2g) and thereby covered the expression domains of *Pt-foxQ2*. At stage 14, *Pt-foxQ2* expression was found in a complex spotted pattern in the brain region and at the base of the labrum (black arrowhead in Fig. 2h). Although it may appear as if expression of *Pt-foxQ2* could be found in the chelicerae (dotted outlines in Fig. 2h), the expression was in fact located underneath the chelicerae and corresponded to the domains located next to the stomodeum at earlier stages (white arrowheads in Fig. 2e, f). The same was true for the complex expression in the neurogenic ectoderm, which is likely only expressed in the neurogenic ectoderm, but not in the epidermal tissue above. The complex expression pattern showed that *Pt-foxQ2* was expressed in the anterior tip of the early germband and later in the neurogenic and non-neurogenic ectoderm and the labrum.

### Downregulation of *Pt-foxQ2* results in labrum defects

In most species, *foxQ2* is downstream of *six3* in the aGRN. In contrast, mutual activation was found in *T. castaneum* indicating a gain of an upstream role in the aGRN (Kitzmann et al. 2017). Therefore, we sought to identify the level of conservation of this genetic interaction between these two transcription factors in the spider*.* There are two paralogs of *six3* in *P. tepidariorum* (*Pt-six3.1* and *Pt-six3.2*) and their embryonic expression patterns had been described previously (Schomburg et al. 2015). At late embryonic stages (stage 10–12), *Pt-six3.1* is expressed in the neurogenic ectoderm and the labrum. Transcripts of *Pt-six3.2* are detected in the labrum, stomodeum and in the anterior median region of the head. At stage 13, *Pt-six3.2* is expressed in the primordia of the lateral eyes and in cells underneath the non-neurogenic ectoderm (Schomburg et al. 2015).

**Fig. 3:**
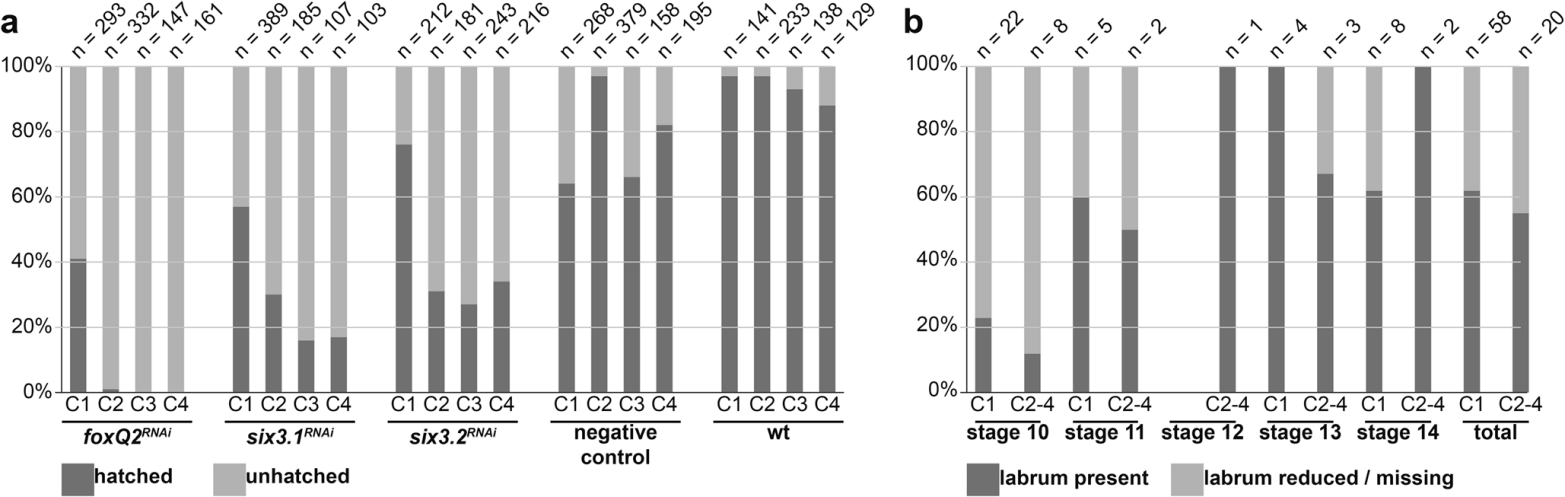
Quantitative analysis of RNAi phenotypes. Overview over hatch rates in cocoons of dsRNA injected female spiders and controls (**a**). Overview of observed labrum defects in *Pt-foxQ2*^*RNAi*^ embryos by developmental stage and cocoons (**b**). Note that cocoons 2–4 showed similar strength (as measured by the hatch rate) and were therefore pooled for the in situ hybridization

**Fig. 4:**
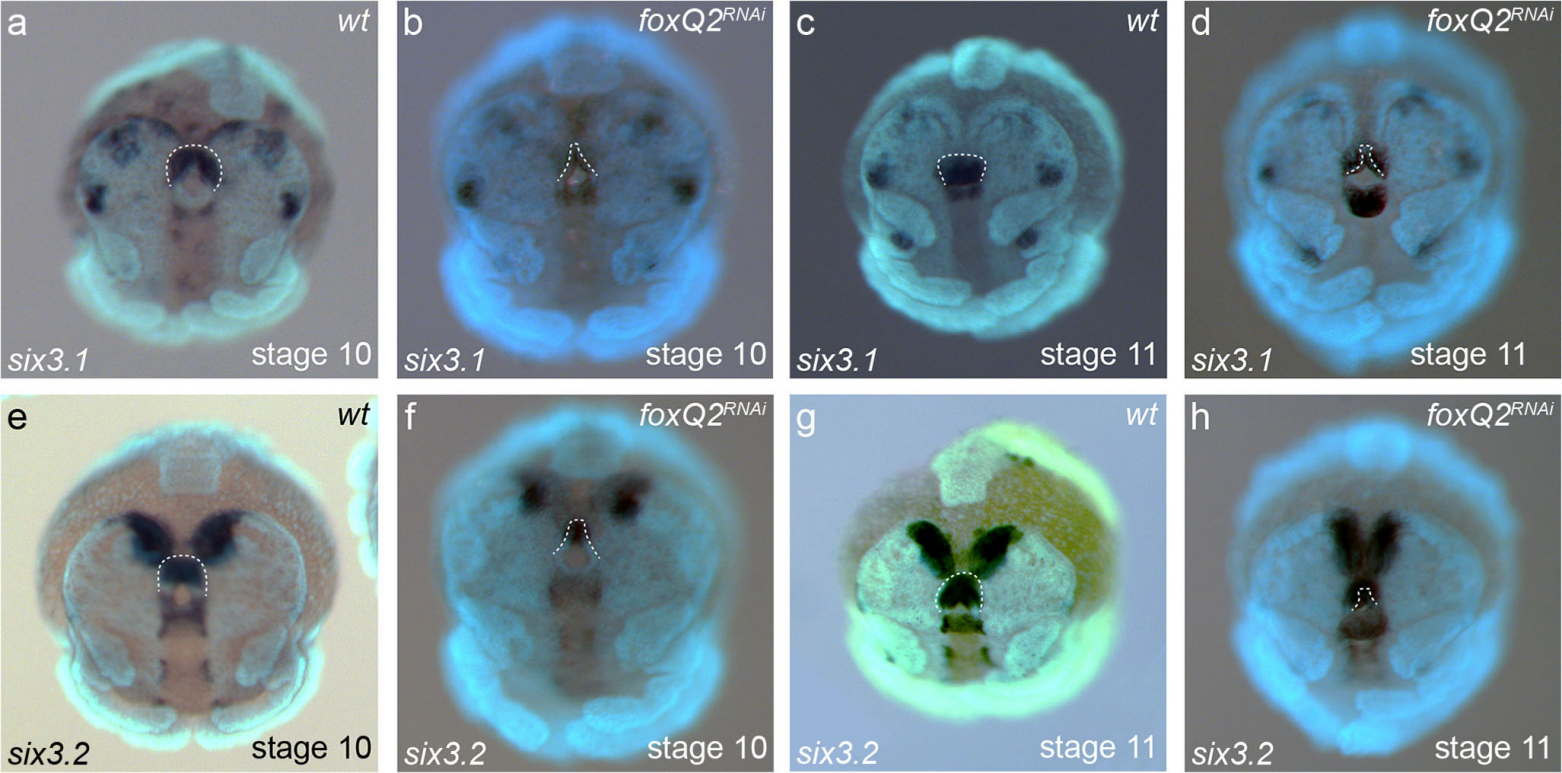
*Pt-six3* expression in *Pt-foxQ2*^*RNAi*^ embryos. Expression of *Pt-six3.1* in *Pt-foxQ2*^*RNAi*^ embryos (**a**–**d**). Expression of *Pt-six3.2* in *Pt-foxQ2*^*RNAi*^ embryos (**e**–**h**). There were no qualitative changes in expression of *Pt-six3* paralogs in *Pt-foxQ2*^*RNAi*^ embryos (note that the overall staining in embryo in b is weaker due to experimental conditions). After *Pt-foxQ2* RNAi, the labrum (dotted lines in all panels) was reduced or missing leading to a secondary reduction of the *Pt-foxQ2*-positive tissue. All embryos shown in frontal view. Developmental stages are indicated in the lower right-hand corner, the stained gene in the lower left-hand corner and treatment in the upper right-hand corner

We performed parental RNAi (pRNAi) to downregulate the expression of *Pt-foxQ2* and analyse the function of this transcription factor during embryogenesis. Likewise, we analysed the function of *Pt-six3.1* and *Pt-six3.2* by RNAi to obtain information about a possible interaction between *Pt-foxQ2* and *Pt-six3.1*/*six3.2*. The analysis included data from females (cocoons 1–4 from 3 different females) that were either injected with dsRNAs (*Pt-foxQ2*, *Pt-six3.1* or *Pt-six3.2*), or with injection buffer (negative control), and from one female spider that was not injected at all (wild type) (supplemental material Tab. S1). Each cocoon was checked for viable and dead embryos (referred to as *Pt-geneX*^RNAi^ embryos) and they were scored for morphological defects. We found that *Pt-foxQ2*^RNAi^, *Pt-six3.1*^RNAi^ and *Pt-six3.2*^RNAi^ embryos showed no cuticle defects. They were able to develop until the embryo started to eclose. However, most of the embryos were unable to hatch from the egg membrane indicating an essential function of those genes. Instead, the membrane remained intact and the embryos stayed at this developmental stage for several days before drying out. Fifty-nine percent of the *Pt-foxQ2*^RNAi^ embryos did not hatch, and the equivalent phenotype was observed for 43% in *Pt-six3.1*^RNAi^, and for 24% in *Pt-six3.2*^RNAi^ embryos in the first cocoon. This phenotype increased in subsequent cocoons (99–100% for *Pt-foxQ2*^RNAi^ embryos, 70–84% for *Pt-six3.1*^RNAi^ embryos, 66–73% for *Pt-six3.2*^RNAi^ embryos) (Fig. 3a). Three to 36% of the negative control embryos did not hatch or were dead (Fig. 3a) which was similar to the rate observed in previous studies (Turetzek et al. 2015). While we did not observe cuticle phenotypes in any of our treatments, we did find morphological differences in the *Pt-foxQ2*^*RNAi*^ embryos at stages 10 (77% in cocoon 1 and 88% in cocoon 2–4) and 11 (40% in cocoon 1 and 50% in cocoon 2–4). In 38% (cocoon 1) and in 45% (cocoon 2–4) of the injected embryos, the labrum was strongly reduced or completely missing (Fig. 3b; Fig. 4b–h).

### Interaction between *Pt-foxQ2*, *Pt-six3.1* and *Pt-six3.2*

In order to test whether *Pt-foxQ2*, *Pt-six3.1* and *Pt-six3.2,* respectively, interact with one another, we performed *Pt-foxQ2* in situ hybridisations in *Pt-six3.1*^RNAi^ and *Pt-six3.2*^RNAi^ embryos and vice versa. We found RNAi against *Pt-foxQ2* had no effect on the expression of either of the *Pt-six3* paralogs, with the exception of the labrum, which normally expresses both *Pt-six3.1* and *Pt-six3.2*, but was missing after *Pt-foxQ2* RNAi (Fig. 4; note that the embryo in b has an overall weaker staining). The morphological defect served as internal control that the RNAi experiment had worked efficiently in these embryos. However, we cannot exclude that the level of downregulation may have been insufficient for an effect on *Pt-six3.1* and *Pt-six3.2* expression.

**Fig. 5:**
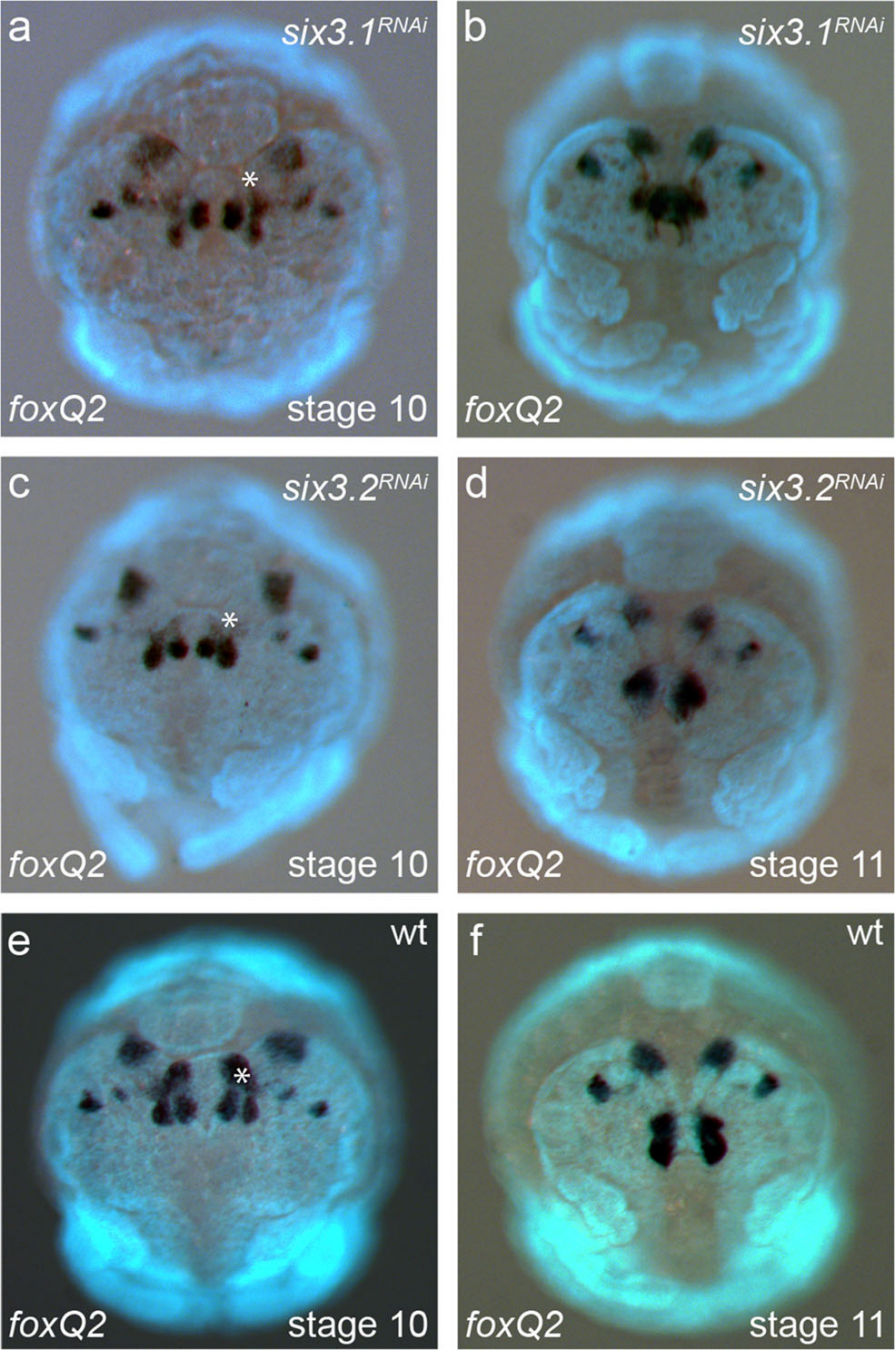
*Pt-foxQ2* expression in *Pt-six3.1/3.2*^*RNAi*^ embryos. Expression of *Pt-foxQ2* in *Pt-six3.1*^*RNAi*^ embryos (**a**, **b**). Expression of *Pt-foxQ2* in *Pt-six3.2*^*RNAi*^ embryos (**c**, **d**). In both treatments, embryos lacked the most anterior expression domain in the anterior median region (asterisks in **a**, **c**) at stage 10. The domain around the stomodeum at stage 11 was reduced (**b**, **d**). Wild-type embryos of stage 10 and 11 are shown for comparison. All embryos shown in frontal view. Developmental stages are indicated in the lower right-hand corner, the stained gene in the lower left-hand corner and treatment in the upper right-hand corner

In the converse experiment, RNAi against neither *Pt-six3.1* nor *Pt-six3.2* showed any apparent morphological differences between wild-type and injected embryos (Fig. 5). The expression of *Pt-foxQ2*, however, was altered. At stage 10, the most anterior of the tripartite median domain (white arrowheads in Fig. 2d) was clearly missing after injection with either *Pt-six3.1* or *Pt-six3.2* dsRNA (asterisks in Fig. 5a, c). At the later stages (Fig. 5b, d), it was difficult to make exact statements about the nature of the changes of expression in the RNAi phenotype, due to the complex dynamic of the expression patterns and the morphological rearrangements during those stages. However, it is clear that the *Pt-foxQ2* expression domain around the stomodeum was reduced in the RNAi experiments, compared to the wild-type staining (white arrowheads in Fig. 2e). The domain was probably also lacking at stage 11, but this was less clear because at that stage, the two domains had fused in wild type (Fig. 2e).

## Discussion

### *Pt-foxQ2* as a conserved anterior marker and important factor in labrum and neural development

Here, we present the first expression and functional study of a *foxQ2* homolog in chelicerates. Like in all metazoan animals analysed so far (Yu et al. 2003; Tu et al. 2006; Yaguchi et al. 2008; Yankura et al. 2010; Range and Wei 2016; Fritzenwanker et al. 2014; Marlow et al. 2014; Santagata et al. 2012; Lee and Frasch 2004; Kitzmann et al. 2017; Sinigaglia et al. 2013; Chevalier et al. 2006), it is expressed as a patterning gene in the aGRN in the early embryo. Later in development, *Pt-foxQ2* is expressed in various regions of the neurogenic ectoderm, which suggests its involvement in neural development might be a conserved aspect among arthropods (Kitzmann et al. 2017; Hunnekuhl and Akam 2014). Furthermore, *Pt-foxQ2* has a similar effect on the development of the labrum as seen in *T. castaneum* (Kitzmann et al. 2017). This hints at a conserved role for *foxQ2* in the development of epidermal head structures in arthropods in contrast to other metazoan lineages where gene knockdown did not reveal strong epidermal defects (Yaguchi et al. 2008; Sinigaglia et al. 2013).

### The two *Pt-six3* paralogs appear to compensate for each other functions in *P. tepidariorum*

Loss of *Pt-six3.1*/*3.2* function in *P. tepidariorum* showed no deleterious effect in the anterior median region in contrast to *T. castaneum*, where this whole region including the labrum is deleted (Posnien et al. 2011). This was unexpected because in all animals studied so far, *six3* acted upstream to or on the same level as *foxQ2*. However, *P. tepidariorum* has two *six3* paralogs with overlapping expression in the anterior median region (compare Fig. 4a and e), which could lead to a compensatory effect. One hint for compensatory function is the elevated portion of hatched animals in both *Pt-six3.1*/*3.2* treatments (about 30% in the second clutch) compared to the *Pt-foxQ2* treatment (1% in the second clutch). Unfortunately, double RNAi has so far not been successful in *P. tepidariorum*.

### An evolutionary playground of anterior interactions

*foxQ2* and *six3* are both prominent factors in anterior development and are commonly co-expressed during embryogenesis and show quite different interactions in different animal species (Fritzenwanker et al. 2014; Hunnekuhl and Akam 2014; Marlow et al. 2014; Martín-Durán and Hejnol 2015; Santagata et al. 2012; Sinigaglia et al. 2013; Tu et al. 2006; Wei et al. 2009; Range and Wei 2016; Kitzmann et al. 2017). In all studied animals but in insects, *six3* is clearly upstream of *foxQ2* in the aGRN. In *T. castaneum*, in contrast, *Tc-foxQ2* gained a more upstream role activating *Tc-six3*. Hence, both genes activate each other and knockdown of either gene leads to similar epidermal phenotypes (i.e. loss of the labrum) (Kitzmann et al. 2017). It had remained unclear whether this interaction was conserved in arthropods or whether it evolved later in the lineages leading to the insects.

As expected, regulation of *Pt-foxQ2* by *Pt-six3.1*/*3.2* was observed in *P. tepidariorum*. However, the RNAi experiments did not provide any evidence for *Pt-foxQ2* being required for *Pt-six3.1*/*3.2* expression as the expression of both *Pt-six3* paralogs remained unchanged in *Pt-foxQ2*^*RNAi*^ embryos, except for a secondary reduction due to the missing tissue of the labrum. We cannot exclude that we do not see an effect on *Pt-six3.1*/*six3.2* expression because the RNAi embryos might still have residual transcripts sufficient for this activation. However, the strong RNAi effect on the labrum argues against this scenario. Hence, assuming that a *Pt-foxQ2* does not activate nor repress *Pt-six3.1*/*3.2* in *P. tepidariorum*, we suggest that the spider situation reflects the ancestral condition (i.e. *six3* upstream of *foxQ2*) while in the lineage leading to the insects *foxQ2* gained a more upstream role by activating *six3* expression.

## Conclusion

**Fig. 6:**
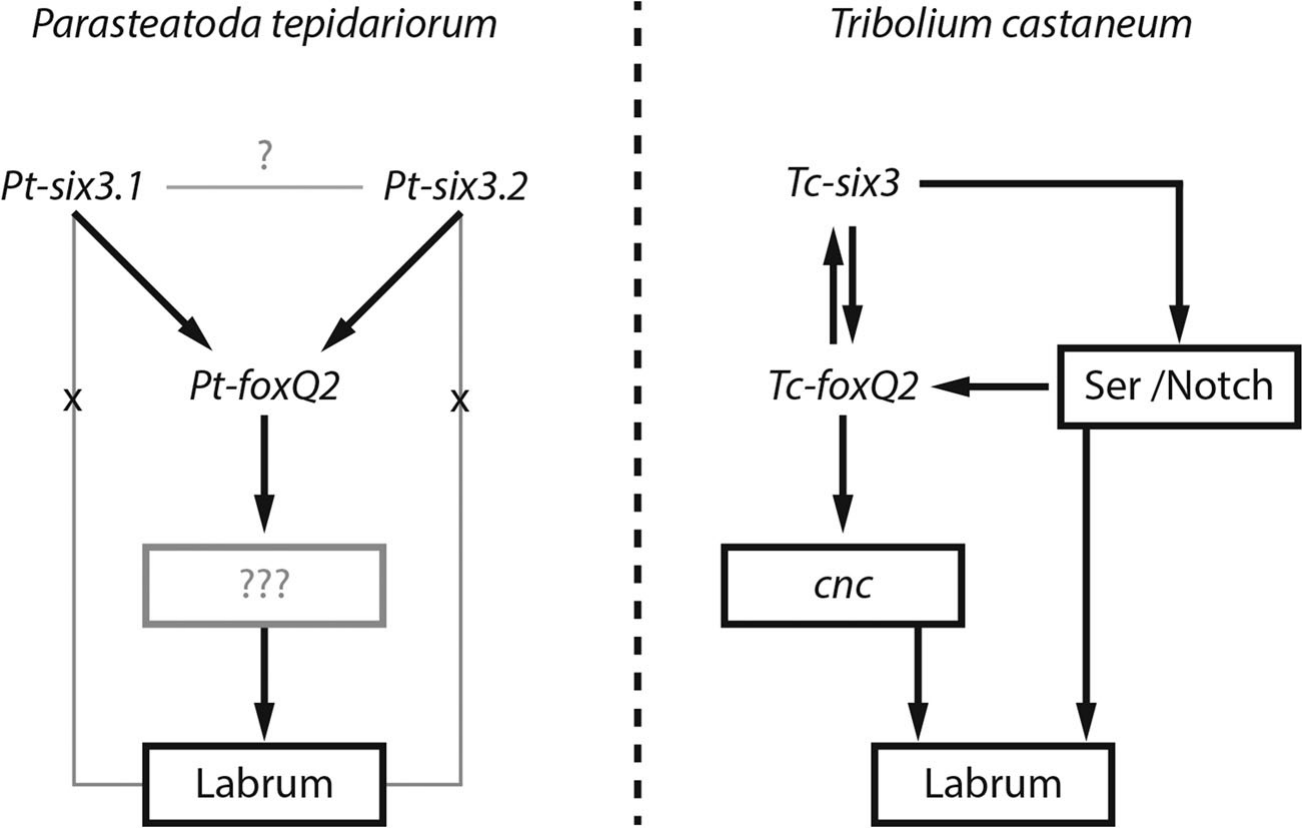
Schematic overview of *Pt-foxQ2* interaction with the *Pt-six3* paralogs. **a** Both *Pt-six3* paralogs are required for proper *Pt-foxQ2* expression while *Pt-foxQ2* RNAi did not lead to alterations in neither of the *Pt-six3* paralogs. These results place *Pt-foxQ2* downstream of *Pt-six3*. Only the knockdown of *Pt-foxQ2* led to morphologically visible reduction or loss of the labrum, while the single RNAis targeting either of the *Pt-six3* paralogs alone did result in no morphological alterations. Further data on labrum development are missing in spiders. **b** The beetle *foxQ2* ortholog, in contrast, is required for proper expression of *Tc-six3* forming a mutual activation loop. Based on data from other species this indicates that *foxQ2* gained a more upstream role in the lineage leading to insects. Interactions from *T. castaneum* are taken from Siemanowski et al. 2015, Kittelmann et al. 2013 and Kitzmann et al. 2017

Our expression analysis adds *Pt-foxQ2* to the list of genes active in the early anterior neurogenic ectoderm in spiders. Our knockdown experiments identify both *Pt-foxQ2* and *Pt-six3.1*/*3.2* as genes required for labrum development in *P. tepidariorum* (Fig. 6). This indicates that the epidermal patterning function of *foxQ2* is an ancestral feature of arthropods. Further, we propose that *Pt-six3.1*/*3.2* is upstream with respect to *Pt-foxQ2* (Fig. 6), although we cannot rule out that *Pt-foxQ2* RNAi knockdown was not strong enough to see an effect on the other genes. We propose that the interactions of the spider aGRN are conserved with those of other animals and suggest that *foxQ2* orthologs gained a more upstream role rather recently in the lineage leading to the insects.

## Electronic supplementary material

ESM 1 (PDF 625 kb)
