# Supplementary material for: six3 acts upstream of foxQ2 in labrum and neural development in the spider Parasteatoda tepidariorum
Source: Dev Genes Evol. 2020 Feb 10;230(2):95–104. doi: 10.1007/s00427-020-00654-9 (PMC7128001; doi:10.1007/s00427-020-00654-9)
Supplement: Supplementary file 1 — (PDF 625 kb) [file 427_2020_654_MOESM1_ESM.pdf]

Supplementary material for:

six3 acts upstream of *foxQ2* in labrum and neural development in the spider *Parasteatoda tepidariorum*

Magdalena Ines Schacht*[1, 2], Christoph Schomburg*[1, 3] and Gregor Bucher[1]

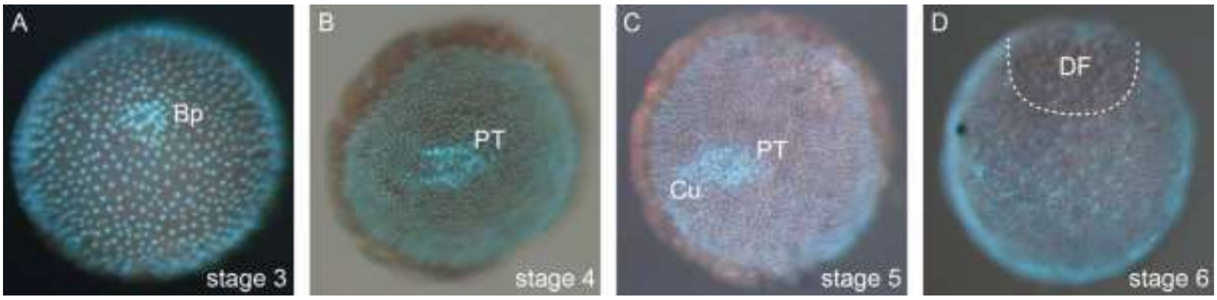

Figure S1. **Early embryonic stages of *P. tepidariorum* devoid of Pt-foxQ2 expression**. (Bp = blastopore, PT = primary thickening, Cu = Cumulus, DF = dorsal field)

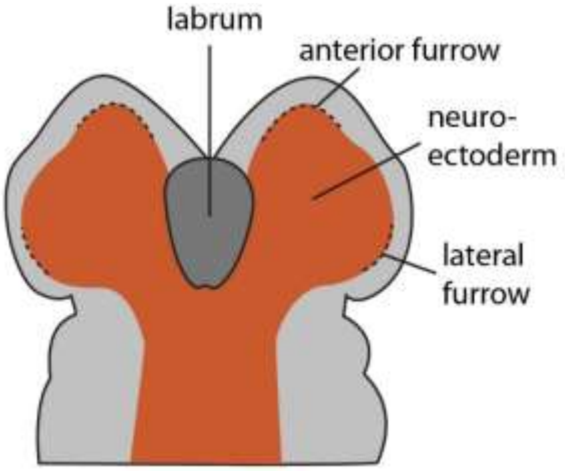

Figure S2. Scheme of stage 11 embryo. Neuroectoderm is shown in orange, labrum is shown in dark grey. The non-neural ectoderm (light grey) of the head folds over the neuroectoderm forming furrows (anterior and lateral furrows). Adapted from Schomburg et al. 2015.

Table S1. Overview over hatching rates in the RNAi experiments and their controls.

foxQ2								
♀	C1		C2		C3		C4	
	hatched	not hatched	hatched	not hatched	hatched	not hatched	hatched	not hatched
**#1**	93	47	2	137	0	59	0	42
**#2**	2	93	0	93	0	30	0	82
**#7**	26	32	0	100	0	58	0	37
	121	172	2	330	0	147	0	161
	293		332		147		161	
	41%	59%	1%	99%	0%	100%	0%	100%

six3.1								
♀	C1		C2		C3		C4	
	hatched	not hatched	hatched	not hatched	hatched	not hatched	hatched	not hatched
**#10**	132	108	7	33	3	37	4	28
**#11**	77	9	14	74	11	21	1	30
**#13**	14	49	35	22	3	32	13	27
	223	166	56	129	17	90	18	85
	389		185		107		103	
	57%	43%	30%	70%	16%	84%	17%	83%

six3.2								
♀	C1		C2		C3		C4	
	hatched	not hatched	hatched	not hatched	hatched	not hatched	hatched	not hatched
**#14**	52	26	10	3	23	24	16	52
**#15**	79	19	5	110	11	110	37	18
**#18**	31	5	41	12	31	44	21	72
	162	50	56	125	65	178	74	142
	212		181		243		216	
	76%	24%	31%	69%	27%	73%	34%	66%

negative control (injection buffer)								
♀	C1		C2		C3		C4	
	hatched	not hatched	hatched	not hatched	hatched	not hatched	hatched	not hatched
**#27**	60	22	116	6	48	13	95	6
**#23**	80	10	123	5	54	2	25	20
**#25**	32	64	128	1	2	39	39	10
	172	96	367	12	104	54	159	36
	268		379		158		195	
	64%	36%	97%	3%	66%	34%	82%	18%

wild type								
♀	C1		C2		C3		C4	
	hatched	not hatched	hatched	not hatched	hatched	not hatched	hatched	not hatched
**wt**	137	4	226	7	128	10	113	16
	141		233		138		129	
	97%	3%	97%	3%	93%	7%	88%	12%
